# QuickStats

**Published:** 2013-01-25

**Authors:** Yelena Gorina

**Figure:**
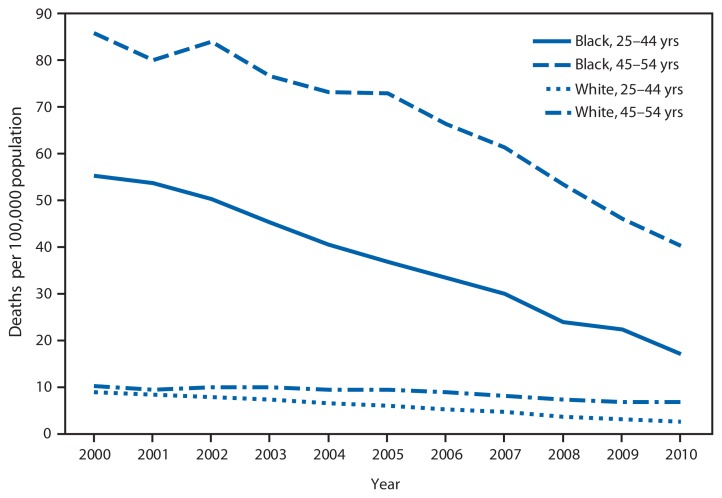
Human Immunodeficiency Virus (HIV) Disease Death Rates* Among Men Aged 25–54 Years, by Race and Age Group — National Vital Statistics System, United States, 2000–2010 * Deaths include those coded as B20–B24 in the *International Classification of Diseases, 10th Revision.*

From 2000 to 2010, HIV disease death rates decreased approximately 70% for both black and white men aged 25–44 years. Rates decreased by 53% for black men aged 45–54 years and 34% for white men aged 45–54 years. Throughout the period, HIV disease death rates for black men were at least six times the rates for white men.

**Sources:** CDC. National Vital Statistics System. Available at http://www.cdc.gov/nchs/data_access/vitalstatsonline.htm.

